# Intrathecal versus local infiltration analgesia for pain control in total joint arthroplasty

**DOI:** 10.1186/s13018-020-01627-4

**Published:** 2020-03-18

**Authors:** Ai-Lan Cai, Sheng-Jie Liu, Bin Wu, Geng Liu

**Affiliations:** Anesthesiology Department, Liaocheng People’s Hospital, No.67 DongChang West Road, Liaocheng, 252000 Shandong China

**Keywords:** Intrathecal analgesia, Local infiltration analgesia, Total joint arthroplasty, Meta-analysis

## Abstract

**Background:**

The purpose of this meta-analysis was to assess the efficacy of intrathecal morphine (ITM) analgesia and local infiltration analgesia (LIA) for pain control in total joint arthroplasty (TJA).

**Methods:**

Embase, PubMed, the Cochrane Library, and Web of Science were systematically searched for randomized controlled trials (RCTs). All RCTs were comparing intrathecal analgesia and local infiltration analgesia in TJA. Primary outcomes were the visual analog scale (VAS) score with rest or mobilization up to 72 h. Secondary outcomes were the total morphine consumption, length of hospital stay, and morphine-related complications.

**Results:**

Compared with the intrathecal analgesia group, the LIA group was associated with a reduction in VAS score with rest up to 72 h. Moreover, LIA was associated with a decrease in VAS score with mobilization at 6 h, 12 h, 48 h, and 72 h. Moreover, LIA significantly reduced total morphine consumption (weighted mean difference (WMD)  = − 15.37, 95% CI − 22.64 to − 8.83, *P*  = 0.000), length of hospital stay (WMD  =  − 1.39, 95% CI − 1.67 to − 1.11, *P*  = 0.000), and morphine-related complications (nausea and pruritus).

**Conclusions:**

Local infiltration provided superior analgesia and morphine-sparing effects within the first 72 h compared with ITM following TJA.

## Introduction

Total joint arthroplasty (TJA) mainly includes total knee arthroplasty (TKA) and total hip arthroplasty (THA). TJA is considered an effective surgical method for the treatment of end-stage osteoarthritis (OA) [1–3]. However, TJA is associated with severe pain after surgery. Effective pain control is crucial for early ambulation and good functional outcomes [4]. Early ambulation can lead to accelerated rehabilitation and a shortened length of hospital stay, which are the essential elements of a fast-track recovery program. Moreover, effective pain control after TJA could not only increase patient satisfaction but also decrease the economic costs caused by the length of hospital stay [5, 6].

Several methods (femoral nerve block [7], intrathecal morphine (ITM) [8], local infiltration analgesia [9], nonsteroidal anti-inflammatory drugs, oral opiates, and gabapentinoids [10]) have been applied in clinical practice for pain control in hip and knee surgeries. Femoral nerve blocks may increase the occurrence of falls and thus have limited clinical use [11]. Oral opiates and gabapentinoids may be associated with complications such as nausea, vomiting, and somnolence [12]. Local infiltration analgesia (LIA) and intrathecal analgesia are two common analgesia methods for pain control in TJA patients. However, it is still inconclusive as to which is preferable for pain relief in TJA. McCarthy et al. [13] concluded that LIA conferred superior analgesia compared with intrathecal morphine at 24 and 48  h following TKA. While Rikalainen-Salmi et al. reported that LIA might only enable early mobilization after THA, it was not associated with less nausea than ITM.

Therefore, we performed a meta-analysis of randomized controlled trials (RCTs) to evaluate the efficiency and safety of LIA and ITM for pain control in TJA.

## Methods

This meta-analysis was based on the recommendations of the Cochrane Handbook for Systematic Reviews of Interventions and was written in accordance with the PRISMA checklist (Preferred Reporting Items for Systematic Reviews and Meta-Analyses).

### Literature search

The following electronic databases were independently and extensively searched by two investigators from their inception through April 2019: Embase, PubMed, the Cochrane Library, and Web of Science. The search keywords were centered on the terms “local infiltration analgesia,” “intrathecal analgesia,” “total knee arthroplasty,” and “total hip arthroplasty,” which were adjusted to each database as necessary. In addition, the bibliographies of the included studies and dissertations were searched for additional publications. The search language was restricted to English. As all analyses were on previously published studies, ethical approval was not necessary.

### Inclusion and exclusion criteria

The PRISMA guidelines were followed for the inclusion of studies in the meta-analysis. The detailed description of the inclusion criteria is as follows: (1) trials had to be properly randomized; (2) no additional agents or interventions confounded the comparison; (3) the patients in the trials were given a bolus dose via local injection; and (4) with respect to trials with several intervention groups, the eligibility of each individual group was evaluated, and only those qualified were included. Early studies published as a series of articles from the same institution or author that contained significant overlapping data were excluded for fear of multiple publication bias. Additionally, case reports, editorials, experimental studies, conference articles, commentaries, and other studies that failed to provide detailed results were excluded.

### Data collection

After duplicates were removed and the study selection process was completed, the titles and abstracts were scanned by two independent investigators. The relevant data were extracted by adopting a predetermined standardized procedure that involved the first authors, year of publication, country, ASA, demographic characteristics of the participants (number of cases, mean age, number of female patients), drug dose of LIA, drug dose of ITM, surgery type, follow-up length, and study type. We attempted to contact the study authors for supplementary information when there were insufficient or missing data in the articles.

### Quality assessment

Two reviewers independently assessed the risk of bias in the RCTs using the Cochrane Collaboration’s tool [14]. The following items were assessed: (i) random sequence generation (selection bias), (ii) allocation concealment (selection bias), (iii) blinding of participants and personnel (performance bias), (iv) blinding of outcome assessment (detection bias), (v) incomplete outcome data (attrition bias), (vi) selective outcome reporting (reporting bias), and (vii) other bias (other bias). Each item was qualified as low risk (L), unclear risk (U), or high risk (H).

### Outcomes

Pain was assessed using the visual analog scale (VAS) pain score (range, 0 [no pain] to 100 [agonizing pain]). A VAS is a measurement instrument used to quantify the amount of pain reported by the patient. Scores can range from 0 (no pain) to 100 (severest pain). We collected VAS scores with rest or mobilization at 6 h, 12 h, 24 h, 48 h, and 72 h, total morphine consumption, length of hospital stay, and the occurrence of nausea, pruritus, and respiratory depression in Microsoft® Excel (Microsoft Corporation, Redmond, WA, USA).

### Statistical analysis

Stata 12.0 (Stata Corp., College Station, TX) was used to perform the meta-analyses. The overall effect size of each anesthetic was calculated as the weighted average of the inverse variance for study-specific estimates. For dichotomous variables, we listed individual and pooled statistics as odds ratios with 95% confidence intervals. For continuous data such as the VAS scores with rest or mobilization at 6 h, 12 h, 24 h, 48 h, and 72 h, total morphine consumption, and length of hospital, we pooled the weighted mean time to union with associated 95% confidence intervals and listed the individual means and standard deviations. Heterogeneity among the individual studies was evaluated based on Cochrane’s *Q* test and the *I*^2^ index, which express, as a percentage, the proportion of variability in the results due to heterogeneity as opposed to sampling error. Considerable heterogeneity was determined when Cochrane’s *Q* test resulted in *P* < 0.10 and *I*^2^ greater than 75%. In such cases, a random effect model was selected for analysis. Otherwise, a fixed effect model was used. If needed, a subgroup analysis was conducted to identify and explain the heterogeneity. A *P* value less than 0.05 was considered significant for all statistical tests.

## Results

### Search results

**Fig. 1:**
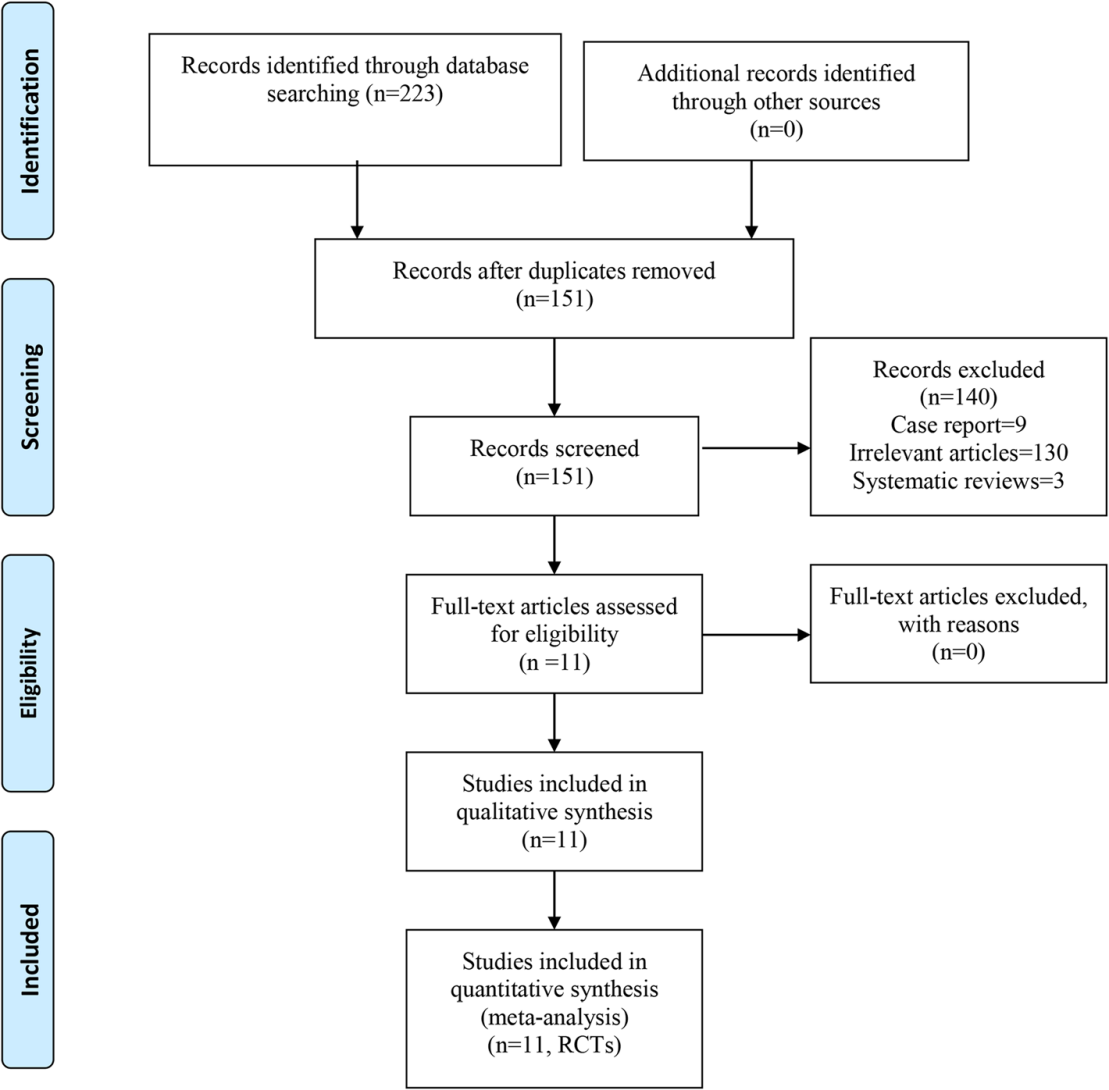
Flow of trials through the meta-analysis

Figure 1 contains a flowchart that describes the process by which we screened and selected trials. The initial literature search yielded 223 articles in all. In addition, a manual search of relevant references did not identify any additional studies. Duplicate checking and title and abstract screening resulted in 72 publications, and the full texts of all 151 articles were available. Among these, 140 were excluded because they were commentaries, 9 were excluded because they were case reports, 130 were excluded owing to being irrelevant studies, and 3 were excluded because they were systematic reviews. Finally, 11 intermediate- to high-quality studies [13, 15–24] were eligible for inclusion in this meta-analysis.

### Characteristics of the trials

The general characteristics of the included studies are shown in Table 1. The publication years ranged from 2007 to 2019. The sample size of the included studies ranged from 15 to 61. The mean age of the included patients ranged from 61 to 71. Three studies compared intrathecal analgesia with LIA in THA patients, and the remaining studies were in TKA patients. All of the included studies were RCTs. All of the included studies performed spinal anesthesia during surgery.

**Table 1:** General characteristic of the included studies

Author	Country	ASA	Cases (*n*, LIA/ITM)	Mean age (years, LIA/ITM)	Female patients (%, LIA/ITM)	Anesthesia	Drug dose of LIA	Drug dose of ITM	Surgery	Follow-up	Study
Andersen et al. [15]	Denmark	I–III	19/21	69/67	52/50	SA	300 mg ropivacaine + 0.5 mg epinephrine	60 mg lidocaine	TKA	72 h	RCT
Andersen et al. [16]	Denmark	I–III	37/38	61/61	38.9/42.4	SA	200 mg ropivacaine + 0.5 mg epinephrine	60 mg lidocaine	THA	14 days	RCT
Binici et al. [17]	Turkey	II–III	15/15	69.4/70.2	41.2/40	SA	240 mg bupivacaine	240 mg bupivacaine	TKA	48 h	RCT
Essving et al. [18]	Sweden	I–III	25/25	71/70	38.9/38.5	SA	300 mg ropivacaine + 0.1 mg epinephrine	0.1 mg morphine	TKA	72 h	RCT
Kuchalik et al. [19]	Sweden	I–III	40/40	66/67	45.4/52.4	SA	300 mg ropivacaine + 0.1 mg epinephrine	0.1 mg morphine	THA	NS	RCT
Rikalainen-Salmi et al. [20]	Finland	I–III	30/30	65/66	NS	SA	120 mg bupivacaine	0.1 mg morphine	THA	72 h	RCT
Spreng et al. [21]	Norway	I–III	33/33	65.8/67.2	49.7/48.4	SA	142.5 ropivacaine	Fentanyl ns	TKA	NS	RCT
Tammachote et al. [22]	Thailand	NS	28/29	69/70	51.2/53.6	SA	100 mg bupivacaine	60 mg bupivacaine + 15 mg epinephrine	TKA	7 days	RCT
Tsukada et al. [23]	Japan	NS	61/50	NS	NS	SA	200 mg ropivacaine + 0.3 mg epinephrine	8 mg morphine	TKA	NS	RCT
Tsukada et al. [24]	Japan	NS	33/37	NS	NS	SA	200 mg ropivacaine + 0.3 mg epinephrine	200 mg ropivacaine + 8 mg morphine	TKA	NS	RCT
McCarthy et al. [13]	Ireland	I–III	22/21	66/64.3	19/43	SA	Levobupivacaine 0.5% 2 mg kg 1 body weight and adrenaline 0.5 mg diluted to a total volume of 100 ml with 0.9% saline	300 μg morphine	TKA	48 h	RCT

*SA* spinal anesthesia, *TKA* total knee arthroplasty, *THA* total hip arthroplasty, *RCT* randomized controlled trials, *NS* not stated

### Risk of bias assessment

**Fig. 2:**
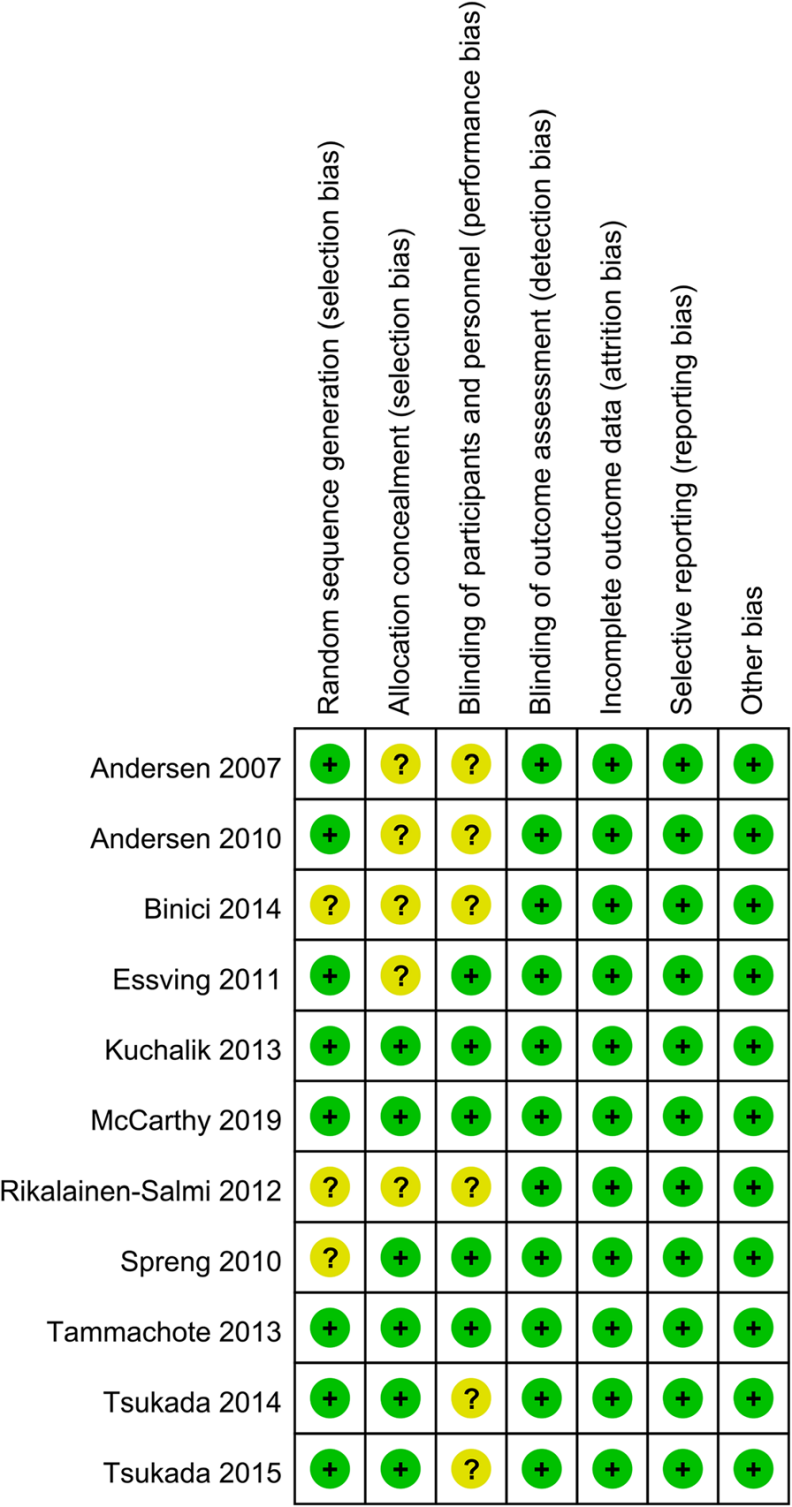
A, the risk of bias summary, +, no bias; −, bias; ?, bias unknown

**Fig. 3:**
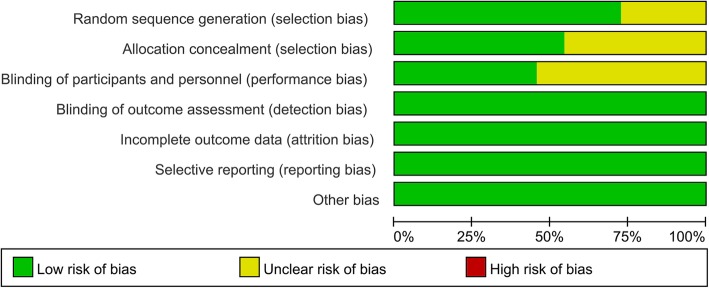
Risk of bias graph of the included randomized controlled trials

The risk of bias summary and risk of bias graph can be seen in Figs. 2 and 3, respectively. Three studies had an unclear risk of bias for random sequence generation, and five studies had an unclear risk of bias for allocation concealment. Six studies had an unclear risk of bias for blinding of participants and personnel. There was a low risk of bias other bias.

### VAS score with rest at 6 h, 12 h, 24 h, 48 h, and 72 h

**Fig. 4:**
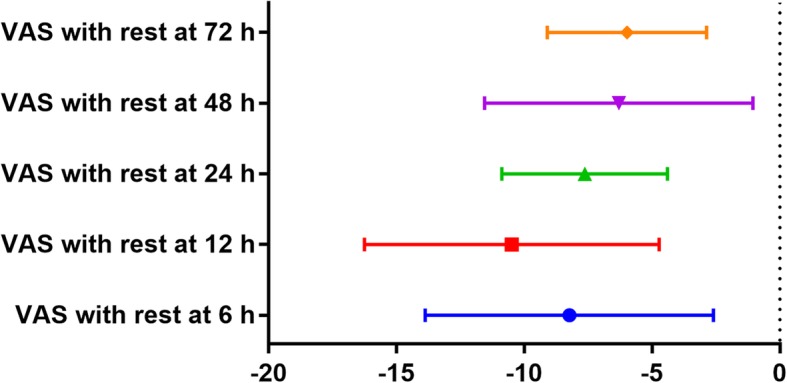
Forest plots of the included studies comparing the VAS scores with rest at 6 h, 12 h, 24 h, 48 h, and 72 h

Eight studies [13, 15, 16, 18–21, 23], with a total of 525 patients, reported the VAS score outcomes 6 h after TJA. A random-effects model was used because significant heterogeneity was found among the studies (*I*^2^  = 98.8%, *P*  =  0.000). The pooled results demonstrated that the LIA group was associated with a reduction in the VAS score with rest at 6 h compared with the ITM group (WMD  =  − 8.23, 95% CI − 13.87 to − 2.59, *P*  = 0.004; Fig. 4).

Compared with the ITM group, the LIA group was associated with a reduction in VAS score with rest at 12 h (WMD  =  − 10.48, 95% CI − 16.25 to − 4.72, *P*  = 0.004; Fig. 4), 24 h (WMD  =  − 7.63, 95% CI − 10.87 to − 4.39, *P*  = 0.000; Fig. 4), 48 h (WMD  =  − 6.30, 95% CI − 11.55 to − 1.05, *P*  = 0.019; Fig. 4), and 72 h (WMD  = − 5.97, 95% CI − 9.09 to − 2.86, *P*  = 0.000; Fig. 4).

### VAS score with mobilization at 6 h, 12 h, 24 h, 48 h, and 72 h

**Fig. 5:**
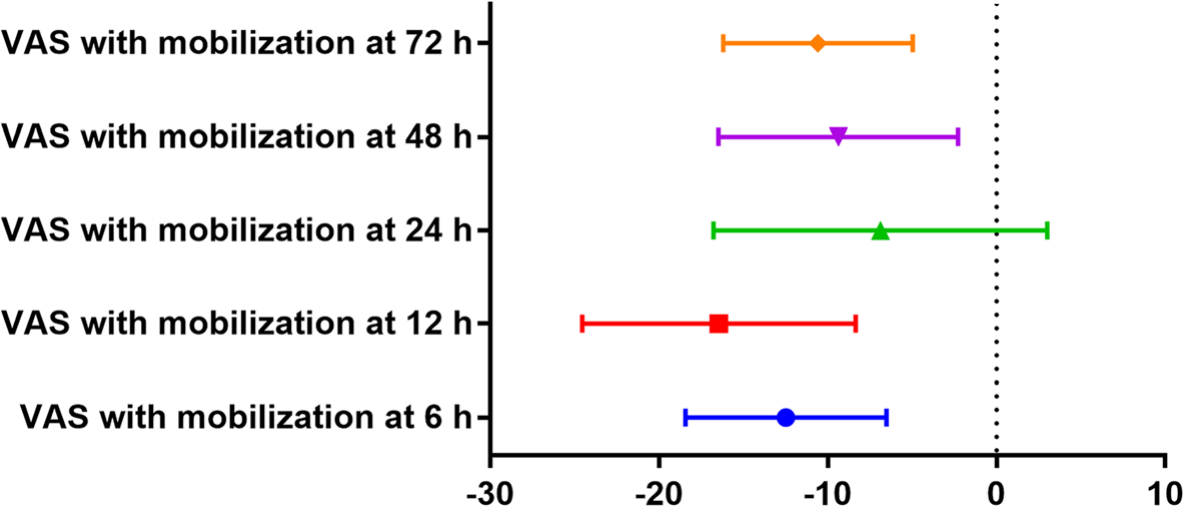
Forest plots of the included studies comparing the VAS scores with mobilization at 6 h, 12 h, 24 h, 48 h, and 72 h

Compared with the ITM group, the LIA group was associated with a reduction in VAS score with mobilization at 6 h (WMD  =  − 12.48, 95% CI − 18.44 to − 6.52, *P*  = 0.000; Fig. 5), 12 h (WMD  =  − 16.45, 95% CI − 24.54 to − 8.35, *P*  = 0.000; Fig. 5), 24 h (WMD  = − 6.88, 95% CI − 16.76 to 3.00, *P*  = 0.172; Fig. 5), 48 h (WMD  = − 9.37, 95% CI − 16.47 to − 2.27, *P*  = 0.010; Fig. 5), and 72 h (WMD  =  − 10.58, 95% CI − 16.19 to − 4.96, *P*  = 0.000; Fig. 5).

### Total morphine consumption

**Fig. 6:**
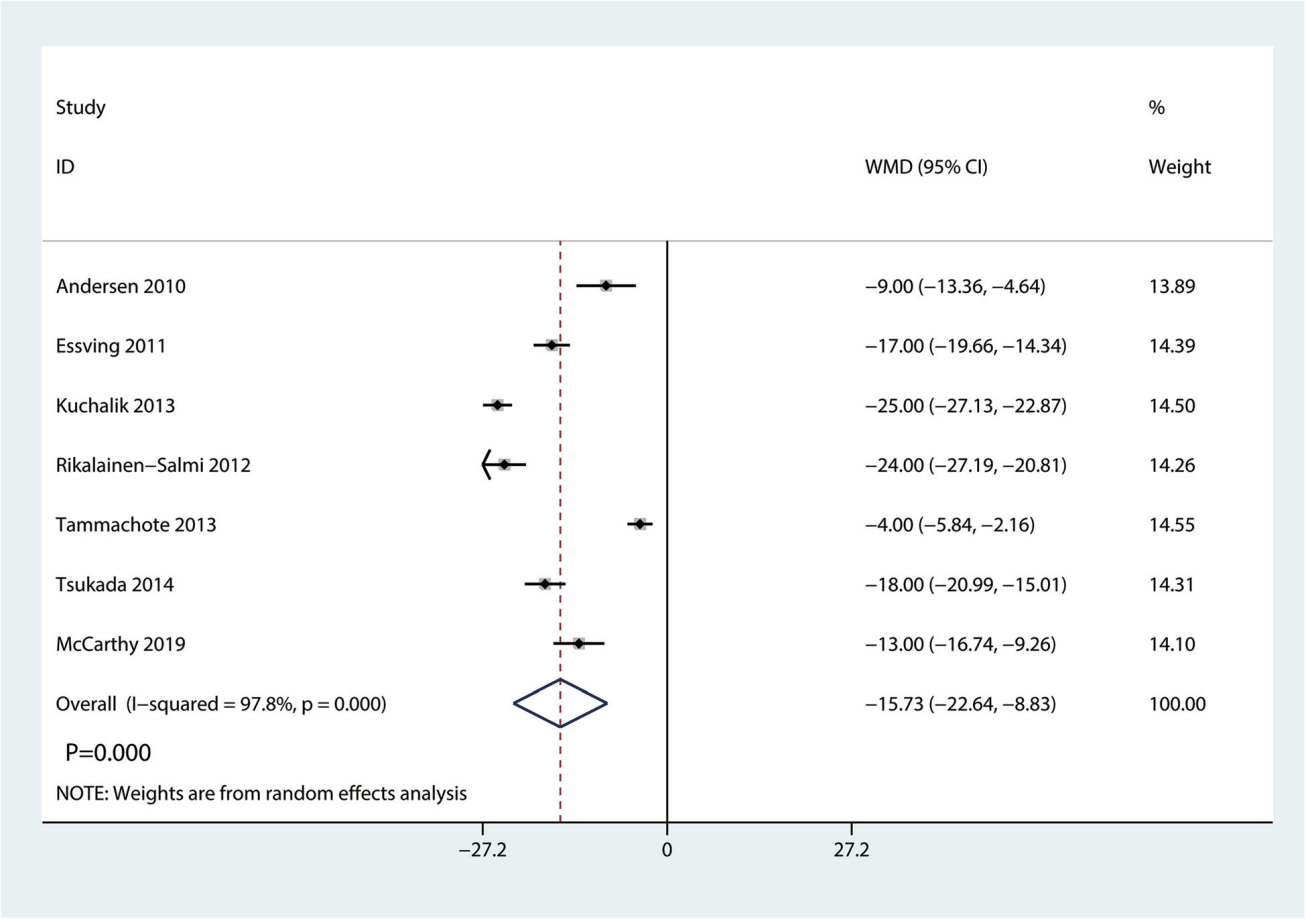
Forest plots of the included studies comparing the total morphine consumption

A total of 7 studies with 439 participants reported the total morphine consumption. Compared with the ITM group, the LIA group was associated with a reduction in total morphine consumption (WMD  = − 15.37, 95% CI − 22.64 to − 8.83, *P*  = 0.000; Fig. 6).

### Length of hospital stay

**Fig. 7:**
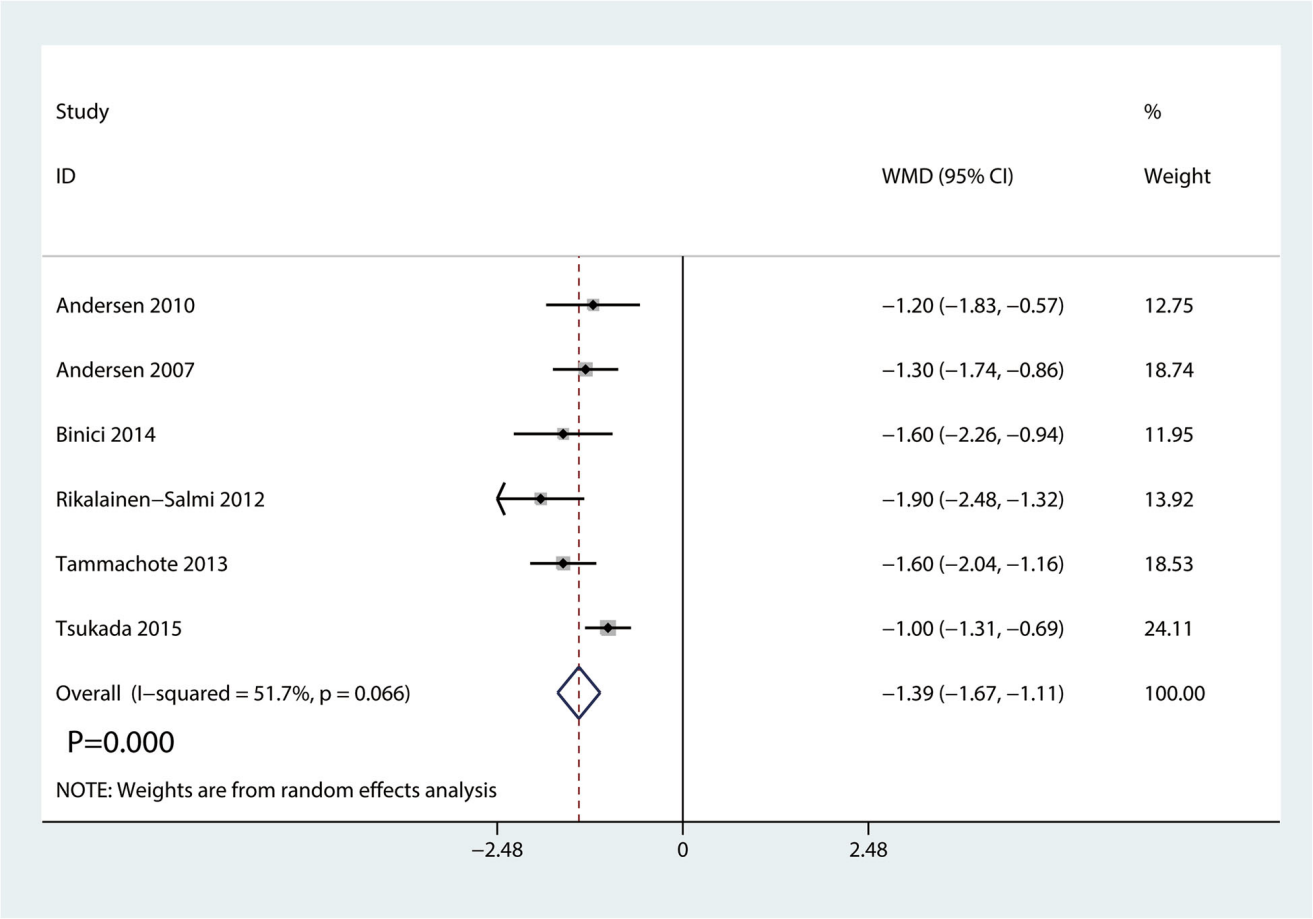
Forest plots of the included studies comparing the length of hospital stay

A total of 7 studies with 332 participants reported the length of hospital stay. Compared with the ITM group, the LIA group was associated with a reduction in the length of hospital stay (WMD  =  − 1.39, 95% CI − 1.67 to − 1.11, *P*  = 0.000; Fig. 7).

### Occurrence of nausea

**Fig. 8:**
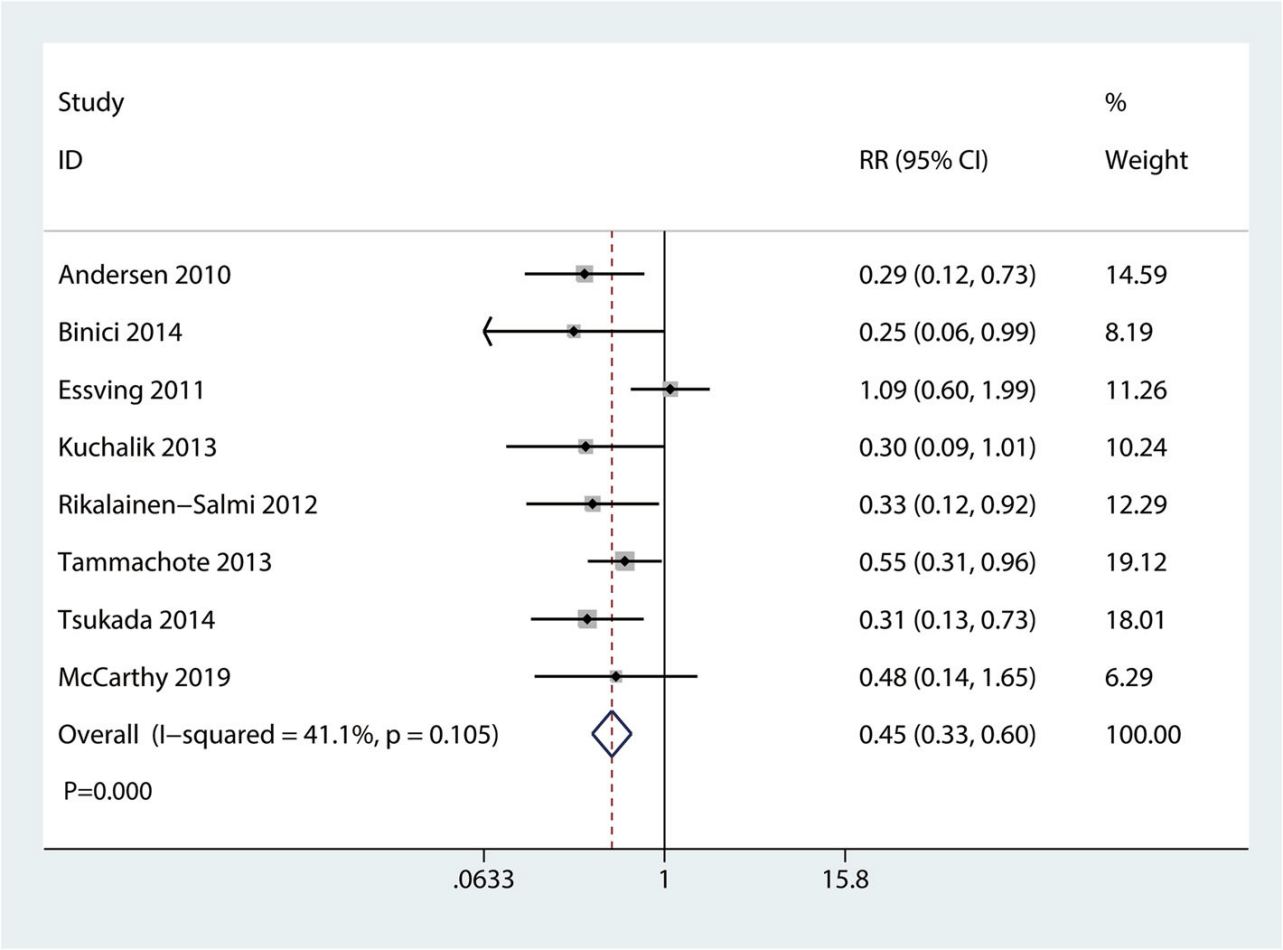
Forest plots of the included studies comparing the occurrence of nausea

A total of 8 studies with 469 participants reported the occurrence of nausea. Compared with the ITM group, the LIA group was associated with a reduction in the occurrence of nausea (RR  =  0.45, 95% CI 0.33 to 0.60, *P*  = 0.000; Fig. 8).

### Occurrence of pruritus

**Fig. 9:**
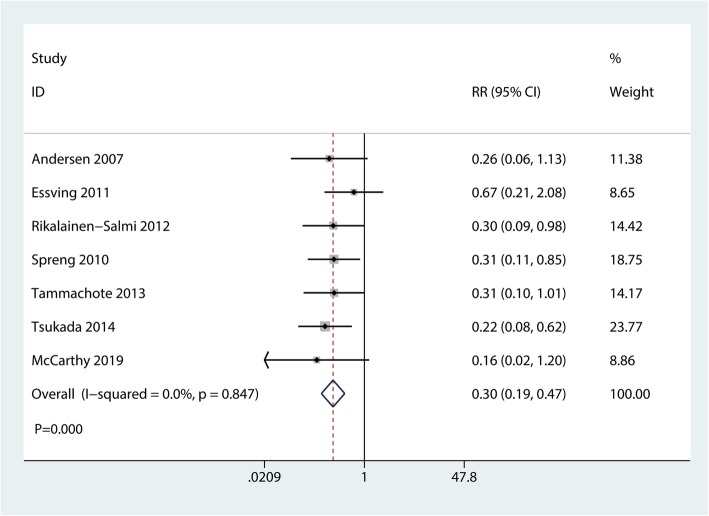
Forest plots of the included studies comparing the occurrence of pruritus

A total of 7 RCTs with 530 participants reported the occurrence of pruritus. Compared with the ITM group, the LIA group was associated with a reduction in the occurrence of pruritus (RR  =  0.30, 95% CI 0.19 to 0.47, *P*  = 0.000; Fig. 9).

### Occurrence of respiratory depression

**Fig. 10:**
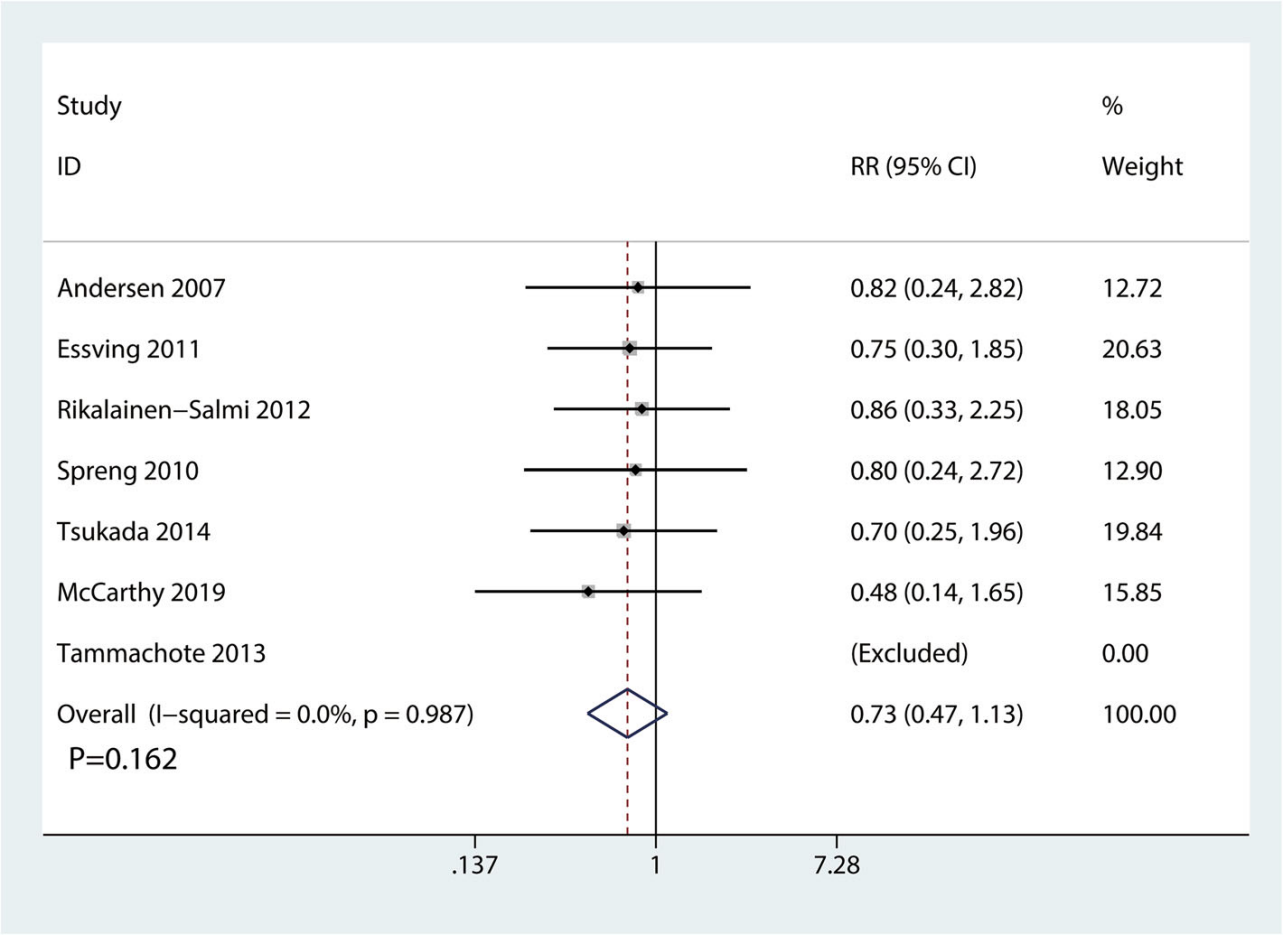
Forest plots of the included studies comparing the occurrence of respiratory depression

A total of 7 RCTs with 460 participants reported the occurrence of respiratory depression. There was no significant difference between the LIA and ITM groups in terms of the occurrence of respiratory depression (RR  =  0.73, 95% CI 0.47 to 1.13, *P*  = 0.162; Fig. 10).

## Discussion

### Main findings

Our meta-analysis found that (1) compared with ITM, LIA significantly reduced pain scores with rest or mobilization at 6 h, 12 h, 24 h, 48 h, and 72 h and (2) LIA further reduced the total morphine consumption, length of hospital stay, and morphine-related complications.

### Comparison with other meta-analyses

Only one relevant meta-analysis has been published [25]. Differences between our meta-analysis and the previous one should be noted. First, the previous meta-analysis included only four trials and 242 patients. In comparison, our current meta-analysis included 11 trials with 382 patients. With the added statistical power of at least 440 cases, our current meta-analysis is the latest and most comprehensive meta-analysis and generally concurs and further reinforces the results from previous meta-analyses. Second, we performed a subgroup analysis and sensitivity analysis. The results from these analyses further confirm our conclusions. Third, we also evaluated the effect of LIA and ITM on the length of hospital stay, which is an important index in clinical practice.

This meta-analysis demonstrated that LIA conferred better analgesia with rest up to 72 h. Moreover, LIA was associated with a reduction in morphine consumption compared with the ITM group. Yin et al. [26] conducted a meta-analysis and found that LIA can be used for patients undergoing THA because of its ability to reduce pain scores and analgesic consumption without any additional adverse events.

The results found that LIA could significantly reduce pain scores with mobilization up to 72 h. Pain with mobilization is more important than pain with rest. LIA is not only effective for pain control but also facilitates patients mobilizing early and returning to normal physiological functions quickly. Jia et al. [25] conducted a meta-analysis of LIA and ITM for total knee and hip arthroplasty. In this meta-analysis, LIA was only superior to ITM for pain control within the first 24 h. A major shortcoming of their meta-analysis was that they only included 4 RCTs with hip and knee surgeries. Lalmand et al. [27] revealed that LIA and ITM have equivalent analgesic effects in elective cesarean delivery. Karlsen et al. [28] conducted a review and found that the pain control efficacy of LIA is equivalent to that of other protocols.

Currently, opioids are commonly administered for pain control after TJA. However, morphine was associated with many insupportable complications, such as nausea and vomiting. Thus, total morphine consumption was also measured as the degree of pain control. We found that LIA was associated with a reduction in morphine consumption compared with ITM. These results were in accordance with the reduction in pain.

We measured morphine-related complications (nausea, pruritus, and respiratory depression) between the LIA and ITM groups. The results found that LIA was associated with a reduction in the occurrence of nausea and pruritus. However, there was no significant difference between the LIA and ITM groups in terms of the occurrence of respiratory depression. Kuchálik et al. [29] revealed that LIA was a safe technique for THA during the long-term follow-up (2 years).

There were a total of 5 limitations in the current meta-analysis. (1) Economic costs and functional outcomes for the LIA and ITM groups were not compared due to insufficient data, and future studies should focus on the economic costs and functional outcomes of LIA and ITM. (2) We included TKA and THA patients, and thus, there was high heterogeneity between the included groups. (3) The dose of anesthetics was different in the included studies, and more studies should be focused on the optimal dose and anesthetic drugs for anesthesia. (4) Long-term follow-ups should be performed to reveal the differences in complications of LIA and ITM.

## Conclusion

Local infiltration provided superior analgesia and morphine-sparing effects within the first 72 h compared with ITM following TJA. There were fewer adverse effects in the local infiltration anesthesia groups. However, it should be noted that these conclusions are based on a limited number of studies and patients. Future studies can focus on the economic costs, functional outcomes, and incidence of adverse events to provide more comprehensive results.

## Data Availability

We state that the data will not be shared since all the raw data are present in the figures included in the article.

## Abbreviations

LIA: Local infiltration analgesia
TJA: Total joint arthroplasty
RCTs: Randomized controlled trials
VAS: Visual analog scale
WMD: Weighted mean difference
TKA: Total knee arthroplasty
THA: Total hip arthroplasty
PRISMA: Preferred Reporting Items for Systematic Reviews and Meta-analyses
L: Low risk
U: Unclear risk
H: High risk
ITM: Intrathecal morphine
